# Association between circulating ECM-associated molecules and cardiovascular outcomes in hemodialysis patients: a multicenter prospective cohort study

**DOI:** 10.1186/s40364-023-00553-x

**Published:** 2024-02-08

**Authors:** Hyeon Seok Hwang, Jihyun Baek, So-Young Lee, Hye Yun Jeong, Jin Sug Kim, Yang Gyun Kim, ShinYoung Ahn, Kipyo Kim, Seok Hui Kang, Min-Jeong Lee, Dong-Young Lee, Yu Ho Lee

**Affiliations:** 1 Division of Nephrology, Department of Internal Medicine, Kyung Hee University Hospital, College of Medicine, Kyung Hee University, Seoul, Korea; 2 Division of Nephrology, Department of Internal Medicine, CHA Bundang Medical Center, CHA University School of Medicine, CHA Bundang Medical Center, 59 Yatap-ro, Bundang-gu, Seongnam-si, Republic of Korea; 3 Department of Biomedical Science, College of Life Science, CHA University, Pocheon, Republic of Korea; 4 Division of Nephrology, Department of Internal Medicine, Kyung Hee University Hospital at Gangdong, College of Medicine, Kyung Hee University, Seoul, Republic of Korea; 5 Division of Nephrology, Department of Internal Medicine, College of Medicine, Korea University, Seoul, Republic of Korea; 6 Division of Nephrology and Hypertension, Department of Internal Medicine, Inha University College of Medicine, Incheon, Republic of Korea; 7 Division of Nephrology, Department of Internal Medicine, College of Medicine, Yeungnam University, Daegu, Republic of Korea; 8 Department of Nephrology, Ajou University School of Medicine, Suwon, Republic of Korea; 9 Division of Nephrology, Department of Internal Medicine, Veterans Health Service Medical Center, Seoul, Republic of Korea

**Keywords:** Extracellular matrix, MMP-2, Biomarker, Hemodialysis, Cardiovascular events

## Abstract

Hemodialysis patients are susceptible to cardiovascular remodeling, which increases the risk of cardiovascular morbidity and mortality. Circulating extracellular matrix (ECM)-associated molecules increase during cardiovascular remodeling and can be potential biomarkers of adverse cardiovascular outcomes. However, their clinical significance in patients undergoing hemodialysis remain unclear. This study aimed to elucidate the association between circulating ECM-associated molecules and cardiovascular outcomes in patients undergoing hemodialysis. To this end, we measured levels of plasma matrix metalloproteinase (MMP)-2, MMP-9, tenascin-C, and thrombospondin-2 in 372 patients with hemodialysis. Plasma MMP-2 levels were significantly higher in patients with future cardiovascular events than in those without future cardiovascular events (*P* = 0.004). All measured molecules had significant correlations with amino-terminal pro-brain natriuretic peptide levels, but the correlation coefficient was the strongest for plasma MMP-2 (rho = 0.317, *P* < 0.001). High plasma MMP-2 levels were predictive of left ventricular (LV) diastolic dysfunction (adjusted odds ratio per a standard deviation increase = 1.48, 95% confidence interval [CI] = 1.05–2.08) and were independently associated with an increased risk of composite cardiovascular events (adjusted hazard ratio per a standard deviation increase = 1.30, 95% CI = 1.04–1.63). In conclusion, high plasma MMP-2 levels are associated with LV diastolic dysfunction and an increased risk of adverse cardiovascular outcomes in hemodialysis patients.

**Supplementary Information:**

The online version contains supplementary material available at 10.1186/s40364-023-00553-x.

## To the editor:

Cardiovascular (CV) disease is the leading cause of mortality in patients with end-stage-kidney disease (ESKD) [1]. In particular, hemodialysis patients are exposed to uremic toxins and hemodynamic stresses, factors that can culminate in pathological CV remodeling and heart failure [2, 3].

The extracellular matrix (ECM) consisted of more than 300 proteins and has a key role in maintaining vascular and myocardial structures [4]. Circulating ECM-associated molecules are indicators of ongoing CV remodeling and can be used as predictors of adverse CV outcomes in general populations [5]. This study investigated the association between circulating ECM-associated molecules and CV outcomes in patients undergoing hemodialysis. `.

We analyzed data from the K-cohort registry, a prospective multicenter cohort comprising Korean hemodialysis patients. Plasma samples were collected from 372 patients, and four ECM-associated proteins were measured: matrix metalloproteinase (MMP)-2, MMP-9, tenascin-C, and thrombospondin-2. The primary outcome was the association between plasma ECM level and composite of cardiac and non-cardiac vascular events. Detailed methods and outcome definitions are available within the Supplemental materials.

Patients with incident CV events had higher levels of plasma MMP-2 among the measured ECM-associated molecules (*P* = 0.004; Supplemental Table 1). Echocardiographic findings indicated that patients who experienced CV events had significantly higher E/E’ values compared to those without CV events (Supplemental Table 2). Furthermore, MMP-2 levels were independently associated with the presence of LV diastolic dysfunction in multivariable logistic regression models (adjusted odd ratio [OR] = 1.48, 95% confidence interval [CI] = 1.05–2.08 per 1 standard deviation [SD] increase, *P* = 0.024; Table 1). In correlation analysis with amino-terminal pro-brain natriuretic peptide (NT-proBNP) levels, the strongest correlation was observed with MMP-2 levels (Rho = 0.317, *P* < 0.001; Supplemental Fig. 1).

**Table 1:** Relationship between circulating ECM-associated molecules and LV dysfunction

	Univariable			Multivariable	
	OR (95% CI)	*P*		OR (95% CI)	*P*
**Diastolic dysfunction**					
MMP-2 per SD	1.96 (1.44–2.67)	< 0.001		1.48 (1.05–2.08)	0.024
MMP-9 per SD	0.98 (0.79–1.23)	0.868		1.05 (0.82–1.34)	0.685
Tenascin-C per SD	1.58 (1.04–2.41)	0.034		1.28 (0.80–2.06)	0.301
Thrombospondin-2 per SD	1.03 (0.99–1.06)	0.172		1.02 (0.98–1.06)	0.442
**LV hypertrophy**					
MMP-2 per SD	1.04 (0.80–1.37)	0.769		0.85 (0.62–1.17)	0.315
MMP-9 per SD	1.05 (0.83–1.34)	0.676		1.11 (0.84–1.45)	0.474
Tenascin-C per SD	1.26 (0.87–1.82)	0.224		1.04 (0.69–1.58)	0.849
Thrombospondin-2 per SD	1.00 (0.97–1.04)	0.994		0.99 (0.95–1.03)	0.563

Multivariable analysis was adjusted for the following covariables: age, sex, body mas index, history of cardiovascular disease, dialysis duration, low density lipoprotein-cholesterol, predialysis systolic blood pressure, and NT-proBNP.

ECM, extraceullar matrix; LV, left ventricular; MMP, matrix metalloproteinase; OR, odds ratio; CI, confidence interval; SD, standard deviation.

A total of 64 (17.2%) composite CV events occurred during a median follow-up of 27.1 months. Of these, 51 (79.7%) and 13 (20.3%) were cardiac and non-cardiac events, respectively. Cox regression analysis showed that patients with high plasma MMP-2 levels showed increased risks of composite CV events after adjusting for relevant clinical parameters (adjusted hazard ratio [HR] = 1.30, 95% CI = 1.04–1.63 per 1 SD increase, *P* = 0.022; Table 2). Its levels also showed independent associations with the occurrence of isolated cardiac events (adjusted HR = 1.43, 95% CI = 1.13–1.82 per 1 SD increase, *P* = 0.003). Circulating MMP-9, tenascin-C, and thrombospondin-2 levels were not predictive of the composite of CV or cardiac events. Plasma MMP-2 levels had a fair discriminative power in distinguishing those at increased risks of composite of CV events, while other biomarkers did not (Supplementary Fig. 2). CV risks were significantly higher in patients with plasma MMP-2 levels exceeding 647.9 ng/mL than in those with lower levels (adjusted HR = 2.14, 95% CI = 1.18–3.89, *P* = 0.012).

**Table 2:** Hazard ratios of ECM level for cardiovascular events

		Univariable				Multivariable	
		HR (95% CI)	*P*			HR (95% CI)	*P*
**Composite of CV events**							
MMP-2	per 1 ng/mL	1.001 (1.000-1.002)	0.056			1.001 (1.000-1.002)	0.039
	per SD	1.27 (1.02–1.59)	0.033			1.30 (1.04–1.63)	0.022
MMP-9	per 1 ng/mL	1.002 (1.000-1.004)	0.107			1.002 (0.999–1.004)	0.152
	per SD	1.12 (0.94–1.33)	0.203			1.12 (0.94–1.32)	0.199
Tenascin-C	per 1 ng/mL	0.989 (0.953–1.027)	0.564			0.982 (0.940–1.026)	0.418
	per SD	0.91 (0.64–1.31)	0.625			0.85 (0.57–1.27)	0.431
Thrombospondin-2	per 1 ng/mL	0.998 (0.982–1.014)	0.799			0.998 (0.981–1.015)	0.822
	per SD	0.99 (0.96–1.03)	0.754			1.00 (0.96–1.03)	0.776
**Cardiac events**							
MMP-2	per 1 ng/mL	1.002 (1.000-1.003)	0.009			1.002 (1.000-1.003)	0.007
	per SD	1.40 (1.11–1.77)	0.004			1.43 (1.13–1.82)	0.003
MMP-9	per 1 ng/mL	1.002 (1.000-1.005)	0.042			1.002 (1.000-1.005)	0.051
	per SD	1.16 (0.97–1.38)	0.097			1.17 (0.98–1.38)	0.078
Tenascin-C	per 1 ng/mL	0.983 (0.942–1.026)	0.443			0.973 (0.926–1.022)	0.278
	per SD	0.85 (0.56–1.28)	0.435			0.78 (0.50–1.21)	0.262
Thrombospondin-2	per 1 ng/mL	0.992 (0.974–1.010)	0.388			0.991 (0.972–1.010)	0.356
	per SD	0.98 (0.94–1.02)	0.364			0.98 (0.94–1.02)	0.333

Multivariable analysis was adjusted for the following covariates: age, sex, body mas index, Charlson comorbidity index, dialysis duration, low density lipoprotein-cholesterol, high sensitivity C-reactive protein, NT-proBNP and spKt/V.

ECM, extracellular matrix; MMP, matrix metalloproteinase; HR, hazard ratio; CI, confidence interval; SD, standard deviation.

MMP families, in particular MMP-2 and − 9, have been implicated in pathologic LV remodeling [6]. Previous studies also demonstrated that tenascin-C and thrombospondin-2 are associated with CV remodeling and could potentially serve as prognostic markers [7, 8]. In this study, we observed independent associations between plasma MMP-2 levels and diastolic dysfunction and incident CV events, while no such associations were found with MMP-9, tenascin-C, and thrombospondin-2 levels. Therefore, MMP-2 may serve as a more useful circulating biomarker for CV outcomes compared to other ECM-associated molecules in hemodialysis patients. In addition, we showed that increased MMP-2 levels are associated with diastolic dysfunction and cardiac events. These findings imply that MMP-2 effectively reflect pathological myocardial remodeling and its detrimental cardiac outcomes. Because NT-proBNP better reflects systolic dysfunction and MMP-2 more accurately indicates diastolic dysfunction, their combined use could enhance prediction of cardiac events in hemodialysis patients.

MMP-9 showed a negative correlation with NT-proBNP (Supplemental Fig. 1). This observation, although not entirely clear, might be attributed to the low prevalence of LV systolic dysfunction (1.3%), which is closely associated with NT-proBNP levels. The relationship between plasma MMP-9 and NT-proBNP warrants further investigation in future studies.

Interestingly, MMP-3 emerged as the sole biomarker in association with adverse CV events in diabetic patients [9]. This suggests that the roles of different MMP subtypes may be disease-dependent.

In conclusion, among the four ECM molecules studied, elevated plasma MMP-2 levels are independently associated with increased risks of LV diastolic dysfunction and adverse CV outcomes in patients undergoing hemodialysis. We anticipate that circulating MMP-2 levels would provide clinicians with opportunities for more accurate CV risk assessment and individualized care.

### Electronic supplementary material

Below is the link to the electronic supplementary material.

Supplementary Material 1

Supplementary Material 2

Supplementary Material 3

Supplementary Material 4

Supplementary Material 5

## Data Availability

The datasets used and/or analyzed during the current study are available from the corresponding author on reasonable request.

## Abbreviations

ESKD: End stage kidney disease
CV: Cardiovascular
ECM: Extracellular matrix
MMP: Matrix metalloproteinase
LV: Left ventricular
E: Peak early diastolic flow velocity
E’: Peak early diastolic tissue velocity
SD: Standard deviation
NT-proBNP: Amino-terminal pro-brain natriuretic peptide
OR: Odds ratio
HR: Hazard ratio
CI: Confidence interval
