# Supplementary material for: Association between circulating ECM-associated molecules and cardiovascular outcomes in hemodialysis patients: a multicenter prospective cohort study
Source: Biomark Res. 2024 Feb 8;12:22. doi: 10.1186/s40364-023-00553-x (PMC10854113; doi:10.1186/s40364-023-00553-x)
Supplement: Supplementary file 1 — Supplementary Material 1 [file 40364_2023_553_MOESM1_ESM.docx]

**SUPPLEMENTARY METHODS:**

**Association between circulating ECM-associated molecules and cardiovascular outcomes in hemodialysis patients: a multicenter prospective cohort study**

**Patient recruitment and data collection**

This study was conducted using data obtained from the K-cohort registry, a multicenter prospective cohort of patients undergoing maintenance hemodialysis in Korea (CRIS No. KCT0003281). The detailed inclusion and exclusion criteria for the K-cohort have been described previously (1). Between June 2016 and March 2020, 637 patients were screened. A total of 372 patients with available whole plasma samples at the time of enrollment were included. The study protocol was approved by the local ethics committee (KHNMC 2016-04-039) and was conducted in accordance with the principles of the Declaration of Helsinki. All participants provided written informed consent before enrollment.

The baseline characteristics and dialysis information of the patients were obtained at the time of inclusion. Information on patient comorbidities was obtained to calculate the Charlson Comorbidity index score (2). Patient monitoring was censored at the time of transfer to peritoneal dialysis or kidney transplantation, loss to follow-up, or withdrawal of patient consent.

**Measurements of plasma ECM-associated molecules**

Plasma samples were collected at the time of study entry, centrifuged for 15 min at 1000 g at room temperature, and stored at -80°C until use. We reviewed previous literature and selected four plasma ECM-associated proteins: MMP-2, MMP-9, tenascin-C, and thrombospondin-2 (3). Enzyme-linked immunosorbent assay (ELISA) was performed using a Magnetic Luminex® Screening Assay multiplex kit (R&D Systems Inc., Minneapolis, MN, USA) to measure these molecules.

**Outcome measures**

The primary outcome was a composite of CV events, including cardiac and non-cardiac vascular events. Cardiac events included acute coronary syndrome, coronary artery disease requiring percutaneous coronary intervention, coronary artery bypass surgery, congestive heart failure, ventricular arrhythmia, cardiac arrest, or sudden death. Non-cardiac events included cerebral infarction, cerebral hemorrhage, and peripheral vascular occlusive diseases that required revascularization or surgical intervention.

**Echocardiographic measures**

Echocardiographic data were obtained from 61.0% (227/372) of the participants enrolled in the study. Echocardiography was performed by experienced cardiologists and trained sonographers at the time of enrolment. Two-dimensional and M-mode echocardiographic data were collected based on the recommendations of the American Society of Echocardiography (4). Left ventricular (LV) end-systolic and end-diastolic diameters, interventricular septal thickness, and LV posterior wall thickness were measured using M-mode echocardiography. The LV end-systolic and end-diastolic volumes, LV ejection fraction, and left atrial dimensions were determined in apical two- and four-chamber views. Peak early diastolic ﬂow velocity (E) and peak late diastolic ﬂow velocity (A) were determined from the mitral valve inﬂow velocity curve using pulsed-wave Doppler. Peak early diastolic tissue velocity (E’) was measured from the septal aspect of the mitral annulus using tissue Doppler. The ratios of the E to A wave (E/A) and E to E’ (E/E’) was calculated. LV systolic dysfunction was defined as LV ejection fraction of lower than 40%, while LV diastolic dysfunction was defined as E/E’ of higher than 15. LV mass was calculated according to the Devereux formula and indexed to height. LV hypertrophy was defined as an LV mass index >115 g/m^2^ in men and >95 g/m^2^ in women (5, 6).

**Statistical analysis**

Data are expressed as means ± standard deviation (SD), median (interquartile range [IQR]), or as the number of patients and percentages. Independent *t*-tests and Chi-square tests were used to compare variables between the groups. ECM-associated molecules were described as medians (IQRs) because these data were non-normally distributed. The correlation between plasma amino-terminal pro-brain natriuretic peptide (NT-proBNP) and ECM-associated molecules was analyzed using Spearman’s analyses. Logistic regression analysis was performed to assess the association between ECM-associated molecules and LV dysfunction. The covariates used for multivariable adjustments were age, sex, body mass index, previous history of CV diseases, dialysis duration, low-density lipoprotein (LDL) cholesterol level, predialysis systolic blood pressure (SBP), and NT-proBNP level. The Cox proportional hazard model was used to identify independent variables associated with CV events. In the multivariable Cox model, the following parameters were included for adjustment: age, sex, BMI, Charlson comorbidity index, dialysis duration, LDL cholesterol, high-sensitivity C-reactive protein, NT-proBNP, and single-pool Kt/V. Receiver operating characteristic (ROC) curves and area under the curve (AUC) values were generated to assess the predictive power of ECM-associated biomarkers to identify those at high risks of CV events. Appropriate cut-off values were determined by Youden’s J statistics. Statistical analyses were performed using SPSS software (version 22.0; IBM Corp., Armonk, NY, USA). *P* values <0.05 were considered statistically signiﬁcant.

**Reference**

1. Hwang HS, Kim JS, Kim YG, Lee SY, Ahn SY, Lee HJ, et al. Circulating PCSK9 Level and Risk of Cardiovascular Events and Death in Hemodialysis Patients. J Clin Med. 2020;9(1).

2. Brusselaers N, Lagergren J. The Charlson Comorbidity Index in Registry-based Research. Methods Inf Med. 2017;56(5):401-6.

3. Trinh K, Julovi SM, Rogers NM. The Role of Matrix Proteins in Cardiac Pathology. Int J Mol Sci. 2022;23(3).

4. Mitchell C, Rahko PS, Blauwet LA, Canaday B, Finstuen JA, Foster MC, et al. Guidelines for Performing a Comprehensive Transthoracic Echocardiographic Examination in Adults: Recommendations from the American Society of Echocardiography. J Am Soc Echocardiogr. 2019;32(1):1-64.

5. Stevens SM, Reinier K, Chugh SS. Increased left ventricular mass as a predictor of sudden cardiac death: is it time to put it to the test? Circ Arrhythm Electrophysiol. 2013;6(1):212-7.

6. Zoccali C, Benedetto FA, Mallamaci F, Tripepi G, Giacone G, Stancanelli B, et al. Left ventricular mass monitoring in the follow-up of dialysis patients: prognostic value of left ventricular hypertrophy progression. Kidney Int. 2004;65(4):1492-8.
