# Supplementary material for: Association between circulating ECM-associated molecules and cardiovascular outcomes in hemodialysis patients: a multicenter prospective cohort study
Source: Biomark Res. 2024 Feb 8;12:22. doi: 10.1186/s40364-023-00553-x (PMC10854113; doi:10.1186/s40364-023-00553-x)
Supplement: Supplementary file 2 — Supplementary Material 2 [file 40364_2023_553_MOESM2_ESM.pptx]

## Slide 1
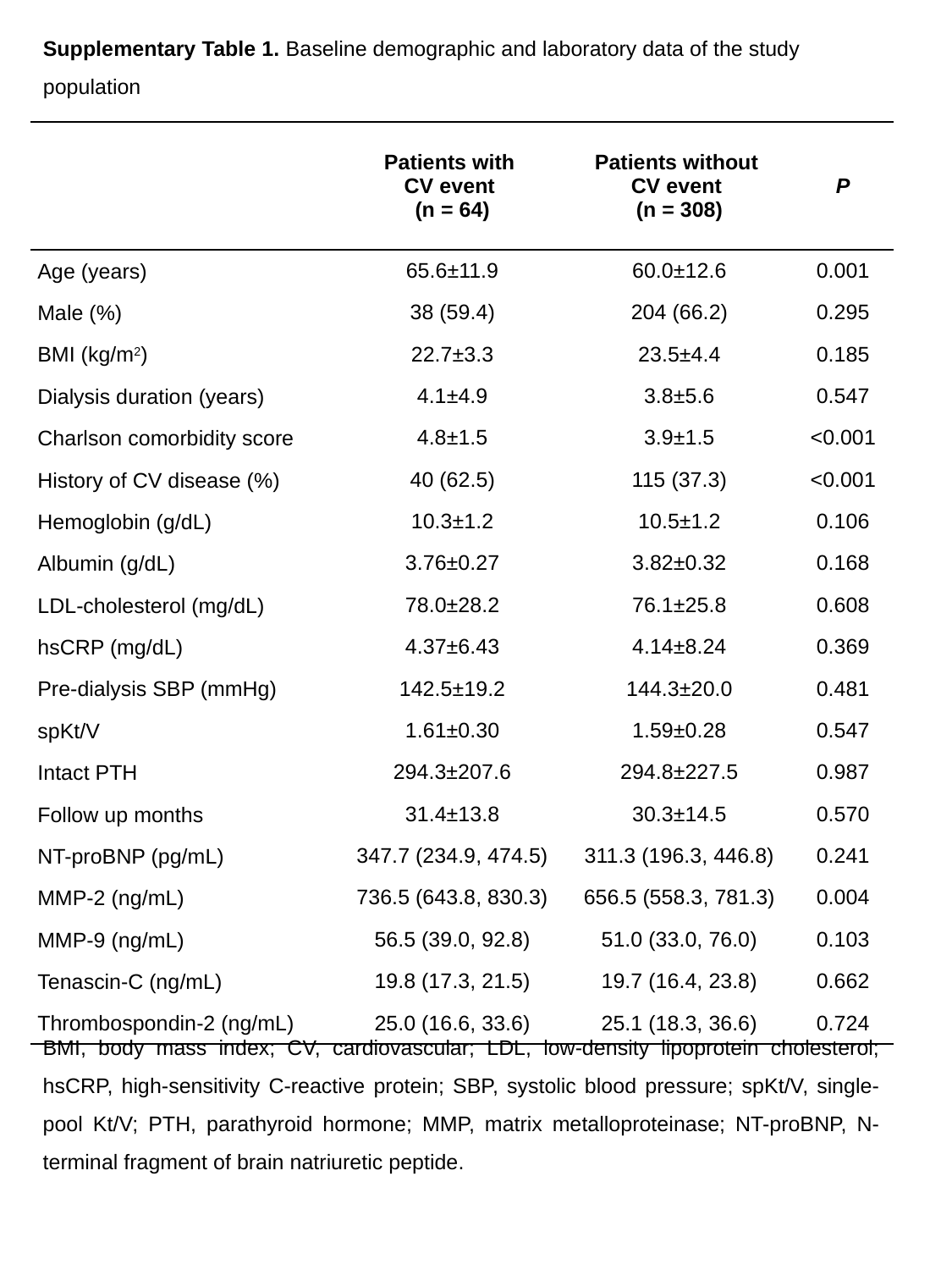

Supplementary Table 1. Baseline demographic and laboratory data of the study population
| | Patients with CV event (n = 64) | Patients without CV event (n = 308) | P |
| --- | --- | --- | --- |
| | | | |
| Age (years) | 65.6±11.9 | 60.0±12.6 | 0.001 |
| Male (%) | 38 (59.4) | 204 (66.2) | 0.295 |
| BMI (kg/m2) | 22.7±3.3 | 23.5±4.4 | 0.185 |
| Dialysis duration (years) | 4.1±4.9 | 3.8±5.6 | 0.547 |
| Charlson comorbidity score | 4.8±1.5 | 3.9±1.5 | <0.001 |
| History of CV disease (%) | 40 (62.5) | 115 (37.3) | <0.001 |
| Hemoglobin (g/dL) | 10.3±1.2 | 10.5±1.2 | 0.106 |
| Albumin (g/dL) | 3.76±0.27 | 3.82±0.32 | 0.168 |
| LDL-cholesterol (mg/dL) | 78.0±28.2 | 76.1±25.8 | 0.608 |
| hsCRP (mg/dL) | 4.37±6.43 | 4.14±8.24 | 0.369 |
| Pre-dialysis SBP (mmHg) | 142.5±19.2 | 144.3±20.0 | 0.481 |
| spKt/V | 1.61±0.30 | 1.59±0.28 | 0.547 |
| Intact PTH | 294.3±207.6 | 294.8±227.5 | 0.987 |
| Follow up months | 31.4±13.8 | 30.3±14.5 | 0.570 |
| NT-proBNP (pg/mL) | 347.7 (234.9, 474.5) | 311.3 (196.3, 446.8) | 0.241 |
| MMP-2 (ng/mL) | 736.5 (643.8, 830.3) | 656.5 (558.3, 781.3) | 0.004 |
| MMP-9 (ng/mL) | 56.5 (39.0, 92.8) | 51.0 (33.0, 76.0) | 0.103 |
| Tenascin-C (ng/mL) | 19.8 (17.3, 21.5) | 19.7 (16.4, 23.8) | 0.662 |
| Thrombospondin-2 (ng/mL) | 25.0 (16.6, 33.6) | 25.1 (18.3, 36.6) | 0.724 |
BMI, body mass index; CV, cardiovascular; LDL, low-density lipoprotein cholesterol; hsCRP, high-sensitivity C-reactive protein; SBP, systolic blood pressure; spKt/V, single-pool Kt/V; PTH, parathyroid hormone; MMP, matrix metalloproteinase; NT-proBNP, N-terminal fragment of brain natriuretic peptide.
