# Supplementary material for: Association between circulating ECM-associated molecules and cardiovascular outcomes in hemodialysis patients: a multicenter prospective cohort study
Source: Biomark Res. 2024 Feb 8;12:22. doi: 10.1186/s40364-023-00553-x (PMC10854113; doi:10.1186/s40364-023-00553-x)
Supplement: Supplementary file 3 — Supplementary Material 3 [file 40364_2023_553_MOESM3_ESM.pptx]

## Slide 1
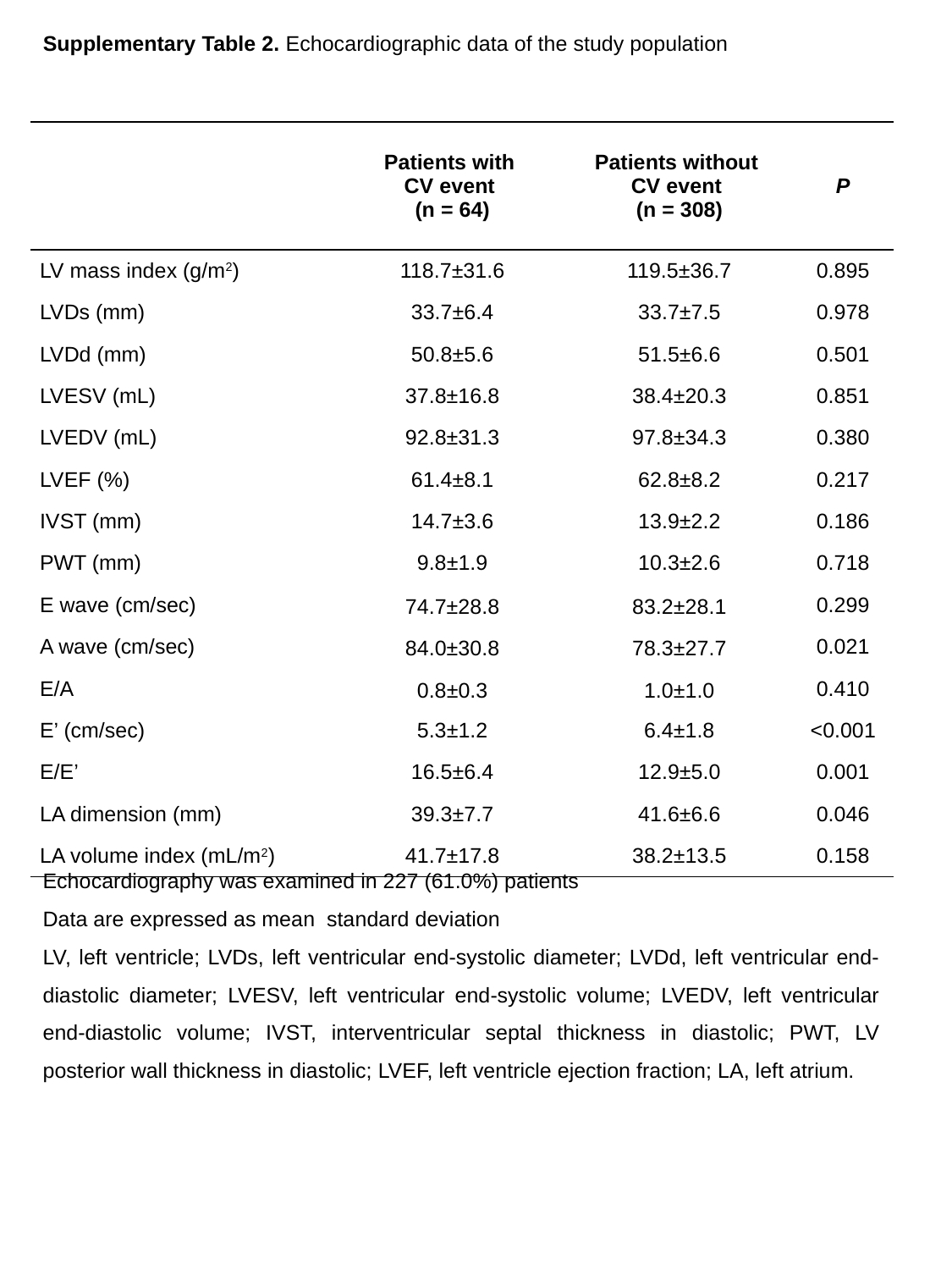

Supplementary Table 2. Echocardiographic data of the study population
| | Patients with CV event (n = 64) | Patients without CV event (n = 308) | P |
| --- | --- | --- | --- |
| | | | |
| LV mass index (g/m2) | 118.7±31.6 | 119.5±36.7 | 0.895 |
| LVDs (mm) | 33.7±6.4 | 33.7±7.5 | 0.978 |
| LVDd (mm) | 50.8±5.6 | 51.5±6.6 | 0.501 |
| LVESV (mL) | 37.8±16.8 | 38.4±20.3 | 0.851 |
| LVEDV (mL) | 92.8±31.3 | 97.8±34.3 | 0.380 |
| LVEF (%) | 61.4±8.1 | 62.8±8.2 | 0.217 |
| IVST (mm) | 14.7±3.6 | 13.9±2.2 | 0.186 |
| PWT (mm) | 9.8±1.9 | 10.3±2.6 | 0.718 |
| E wave (cm/sec) | 74.7±28.8 | 83.2±28.1 | 0.299 |
| A wave (cm/sec) | 84.0±30.8 | 78.3±27.7 | 0.021 |
| E/A | 0.8±0.3 | 1.0±1.0 | 0.410 |
| E’ (cm/sec) | 5.3±1.2 | 6.4±1.8 | <0.001 |
| E/E’ | 16.5±6.4 | 12.9±5.0 | 0.001 |
| LA dimension (mm) | 39.3±7.7 | 41.6±6.6 | 0.046 |
| LA volume index (mL/m2) | 41.7±17.8 | 38.2±13.5 | 0.158 |
