# Supplementary material for: Association between circulating ECM-associated molecules and cardiovascular outcomes in hemodialysis patients: a multicenter prospective cohort study
Source: Biomark Res. 2024 Feb 8;12:22. doi: 10.1186/s40364-023-00553-x (PMC10854113; doi:10.1186/s40364-023-00553-x)
Supplement: Supplementary file 4 — Supplementary Material 4 [file 40364_2023_553_MOESM4_ESM.pptx]

## Slide 1
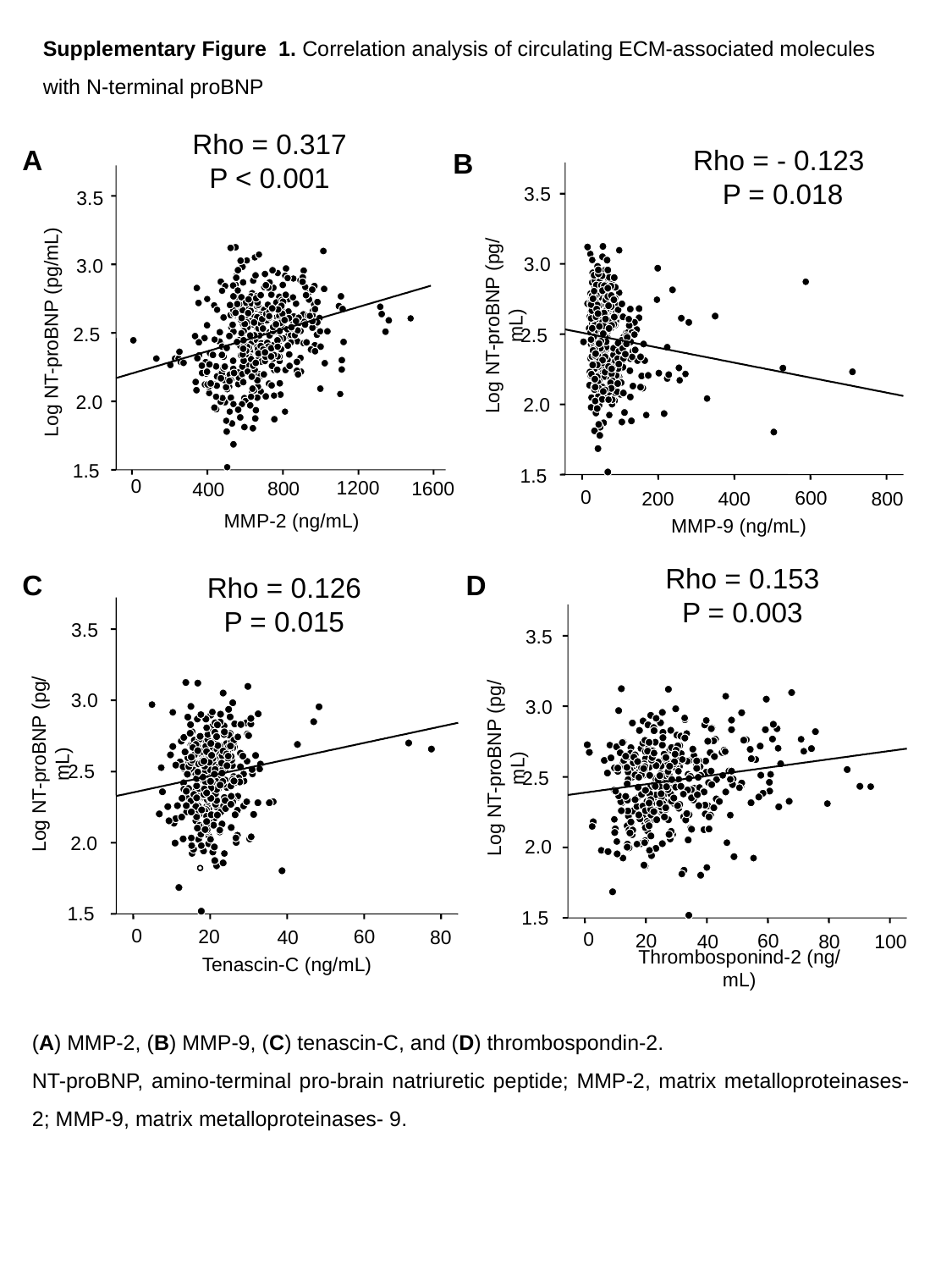

Supplementary Figure 1. Correlation analysis of circulating ECM-associated molecules with N-terminal proBNP
Rho = 0.317
P < 0.001
A
Rho = - 0.123
P = 0.018
B
3.5
3.0
Log NT-proBNP (pg/mL)
2.5
2.0
1.5
0
600
200
800
400
MMP-9 (ng/mL)
3.5
3.0
Log NT-proBNP (pg/mL)
2.5
2.0
1.5
0
1200
400
1600
800
MMP-2 (ng/mL)
Rho = 0.153
P = 0.003
D
C
Rho = 0.126
P = 0.015
3.5
3.0
Log NT-proBNP (pg/mL)
2.5
2.0
1.5
0
60
20
80
40
Tenascin-C (ng/mL)
3.5
3.0
Log NT-proBNP (pg/mL)
2.5
2.0
1.5
0
60
20
100
80
40
Thrombosponind-2 (ng/mL)
(A) MMP-2, (B) MMP-9, (C) tenascin-C, and (D) thrombospondin-2.
NT-proBNP, amino-terminal pro-brain natriuretic peptide; MMP-2, matrix metalloproteinases-2; MMP-9, matrix metalloproteinases- 9.
