# Supplementary material for: Association between circulating ECM-associated molecules and cardiovascular outcomes in hemodialysis patients: a multicenter prospective cohort study
Source: Biomark Res. 2024 Feb 8;12:22. doi: 10.1186/s40364-023-00553-x (PMC10854113; doi:10.1186/s40364-023-00553-x)
Supplement: Supplementary file 5 — Supplementary Material 5 [file 40364_2023_553_MOESM5_ESM.pptx]

## Slide 1
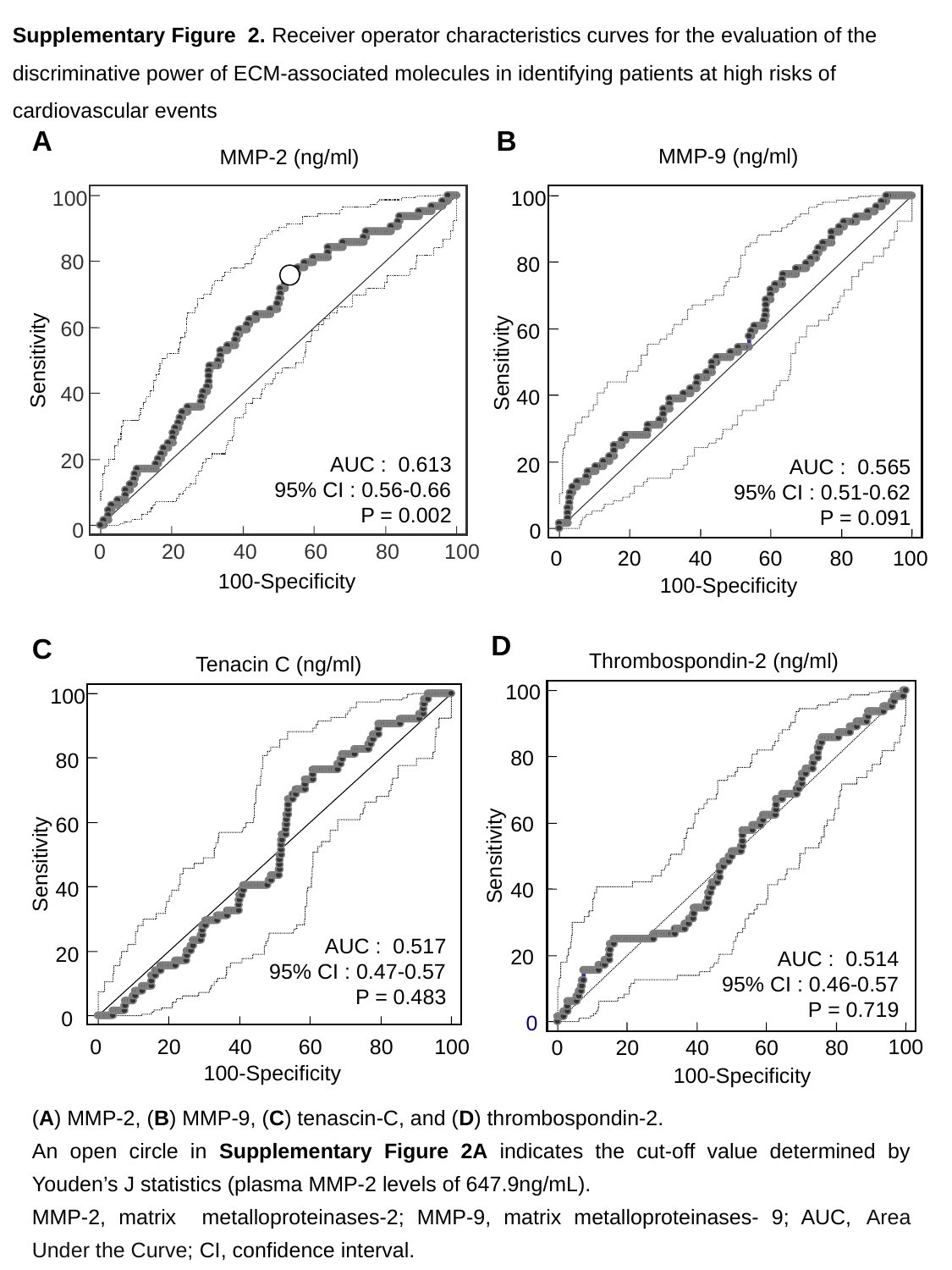

Supplementary Figure 2. Receiver operator characteristics curves for the evaluation of the discriminative power of ECM-associated molecules in identifying patients at high risks of cardiovascular events
A
B
MMP-9 (ng/ml)
MMP-2 (ng/ml)
100
80
60
Sensitivity
40
20
0
0
20
40
60
80
100
100-Specificity
100
80
60
Sensitivity
40
20
0
0
20
40
60
80
100
100-Specificity
D
C
Thrombospondin-2 (ng/ml)
Tenacin C (ng/ml)
100
80
60
Sensitivity
40
20
0
100
0
20
40
60
80
100-Specificity
100
80
60
Sensitivity
40
20
0
0
20
40
60
80
100
100-Specificity
AUC : 0.613
95% CI : 0.56-0.66
P = 0.002
AUC : 0.565
95% CI : 0.51-0.62
P = 0.091
AUC : 0.517
95% CI : 0.47-0.57
P = 0.483
AUC : 0.514
95% CI : 0.46-0.57
P = 0.719
(A) MMP-2, (B) MMP-9, (C) tenascin-C, and (D) thrombospondin-2.
An open circle in Supplementary Figure 2A indicates the cut-off value determined by Youden’s J statistics (plasma MMP-2 levels of 647.9ng/mL).
MMP-2, matrix metalloproteinases-2; MMP-9, matrix metalloproteinases- 9; AUC, Area Under the Curve; CI, confidence interval.
